# An Energy Efficient Load Balancing Tree-Based Data Aggregation Scheme for Grid-Based Wireless Sensor Networks

**DOI:** 10.3390/s22239303

**Published:** 2022-11-29

**Authors:** Neng-Chung Wang, Chao-Yang Lee, Young-Long Chen, Ching-Mu Chen, Zi-Zhen Chen

**Affiliations:** 1 Department of Computer Science and Information Engineering, National United University, Miaoli 360302, Taiwan; 2 Department of Aeronautical Engineering, National Formosa University, Yunlin 632301, Taiwan; 3 Department of Computer Science and Information Engineering, National Taichung University of Science and Technology, Taichung 404336, Taiwan; 4 Department of Electrical Engineering, I-NING High School, Taichung 407001, Taiwan

**Keywords:** data aggregation, grid, load balancing, tree, wireless sensor network

## Abstract

A wireless sensor network (WSN) consists of a very large number of sensors which are deployed in the specific area of interest. A sensor is an electronic device equipped with a small processor and has a small-capacity memory. The WSN has the functions of low cost, easy deployment, and random reconfiguration. In this paper, an energy-efficient load balancing tree-based data aggregation scheme (LB-TBDAS) for grid-based WSNs is proposed. In this scheme, the sensing area is partitioned into many cells of a grid and then the sensor node with the maximum residual energy is elected to be the cell head in each cell. Then, the tree-like path is established by using the minimum spanning tree algorithm. In the tree construction, it must meet the three constraints, which are the minimum energy consumption spanning tree, the network depth, and the maximum number of child nodes. In the data transmission process, the cell head is responsible for collecting the sensing data in each cell, and the collected data are transmitted along the tree-like path to the base station (BS). Simulation results show that the total energy consumption of LB-TBDAS is significantly less than that of GB-PEDAP and PEDAP. Compared to GB-PEDAP and PEDAP, the proposed LB-TBDAS extends the network lifetime by more than 100%. The proposed LB-TBDAS can avoid excessive energy consumption of sensor nodes during multi-hop data transmission and can also avoid the hotspot problem of WSNs.

## 1. Introduction

In a wireless sensor network (WSN), a sensor node collects and aggregates the sensing data, and then transmits the data to the base station (BS). Due to the advancement of electronic technology, there are more and more applications combining sensors and wireless network technology [1,2,3]. Sensors are often used as temperature sensors, infrared sensors, and carbon dioxide sensors. Due to the small size and low price of sensors, sensors can be deployed in a large number in specific sensing environments to detect and obtain useful data. The technology of WSNs can be applied to the related detection of military battlefields, such as poison gas, human body temperature, and other related detection applications. Currently, it is widely used in smart home and medical care detection, along with other applications such as traffic control, health monitoring, and industrial control [4,5,6].

In a WSN, a very large number of sensors are deployed in a specific network environment, and the amount of data collected by the sensors is getting larger and larger. During the process of data transmission, some sensors may have too much energy consumption while transmitting and receiving data. Because wireless sensors are energy-constrained, battery replacement and maintenance management may be difficult in certain environments. Therefore, energy-efficient algorithms could reduce energy consumption so that sensors can continue to operate to extend the network lifetime.

The main contribution of the paper is to propose a load balancing tree-based data aggregation scheme (LB-TBDAS), which restricts the tree structure in grid-based WSNs. The tree structure of LB-TBDAS must meet three restrictions: minimum energy consumption spanning tree, network depth, and a maximum number of child nodes to make the energy consumption of sensor nodes uniform.

The rest of this paper is organized as follows. First, Section 1 introduces the basic information of this paper. In Section 2, we review the background of related work. Section 3 describes the proposed scheme. In Section 4, the simulation results are discussed. Finally, Section 5 gives some conclusions.

## 2. Related Work

In recent years, many WSN research studies have proposed various data aggregation schemes. The diffusion-based data aggregation scheme is data-centric in that each sensor node is not distinguished by address but is addressed by the sensed data. When collecting data, the user sends a request that will spread out through other sensor nodes in WSNs. If the sensor node repeatedly receives the same data, the data are ignored. When the sensor node has data that satisfies the request, the sensor node transmits the data to the BS through the reverse path.

The Direct Diffusion [7] uses adjacent sensor nodes to exchange the information with each other. In this scheme, it is very suitable for use in dynamic networks for searching data in real time. The disadvantage is that the diffusion data are easily affected by the distance of the BS. PEGASIS [8] is a data aggregation scheme based on a chain structure and uses the greedy algorithm to build a chain structure. During each round of data transmission, the sensor nodes on both sides of the chain transmit data in the direction of the chain head through the adjacent sensor nodes. Each receiving sensor node aggregates the data and then transmits the data to the BS via the chain head. This scheme reduces the energy consumption of sensor nodes to form a dynamic cluster. Only the chain head is required to transmit data to the BS. Even if the range of the WSN is expanded, it can still have good performance. In addition, the nodes take turns serving as the chain head responsible for sending the aggregated data to the BS, which balances the energy consumption of sensing nodes. CCRS [9] presented a concentric clustering scheme that combines the cluster structure and the chain structure to reduce the energy consumption of PEGASIS in each round. According to the strength of the signal transmitted by the BS, it assigns its own level, and uses the greedy algorithm. The chain structure of each level is established, and the chain head takes turns to serve. TTDD [10] is a grid-based scheme with dissemination nodes responsible for storing and transmitting the sensing data. The data collector, called sink, transmits the query message to its neighboring sensor nodes directly, and the nodes forward the sensing data in turn toward the source node. During the process of query message forwarding, path information to the data collector is established so that the requested data are transmitted from the source to the sink. MLDAT [11] is a multi-level data aggregation technique for WSNs. In the scheme, the sensor preprocesses the sensing data, filters the raw data, and removes the redundant data to achieve energy saving of the WSN. This scheme reduces the transmission latency and packet retransmission, thereby improving bandwidth utilization. HDAA [12] is a hybrid data aggregation algorithm for WSNs. In this scheme, the sensor provides active data processing in real time through an enhanced aggregation technique to eliminate the duplication and unwanted data. This scheme reduces the energy consumption and communication delay, thereby extending network lifetime.

The tree-based data aggregation scheme is the data aggregation scheme using a tree-like path for data transmission. In the tree structure, the parent node is responsible for data collection of the child nodes, and the sensor node transmits the data to the parent node. Then, the data of the child nodes are aggregated in the parent node and then the aggregated data are transmitted to the upper layer to form a hierarchical structure. E-Span [13] uses a decentralized protocol to construct an energy-aware spanning tree. The node with the highest remaining energy is found from all the sensor nodes as the root node. The selection of parent nodes of other nodes is based on the energy of all neighboring nodes and the distance between these nodes and the root node. TBEEP [14] is a tier-based energy-efficient protocol. In the scheme, the sensing area is divided into three layers based on the distance between the sensor node and the base station, and then a tree structure is constructed by using Prim’s algorithm. In the scheme, a minimum spanning tree is generated in each round and the load is properly balanced in the network. This scheme increases the lifetime of the network by increasing the number of rounds. EPDA [15] is an energy-efficient and privacy-preserving data aggregation algorithm. In the scheme, an aggregation tree is built in the WSN, and then the leaf nodes of the tree are connected to form multiple chains. This scheme only needs to slice the data perceived by the chain tail node to ensure privacy. This scheme significantly decreases energy consumption and extends the lifetime of the network. PEDAP [16] uses a tree-like structure for a data aggregation protocol based on the minimum spanning tree. When establishing the tree structure of PEDAP, the energy consumption between sensor nodes is calculated to determine the minimum spanning tree for data transmission. During each round of data transmission, the sensor node at the bottom of the tree transmits the sensing data to its parent node and aggregates the data, and then repeats the same process to transmit the data towards the BS. The advantage of PEDAP is that the energy consumption during data transmission between the sensor nodes is minimized to achieve the data transmission with the lowest energy consumption. GB-PEDAP [17] is a grid-based tree-structured data aggregation scheme. This scheme divides the sensing area into many cells of a grid and each cell has a cell head as the relay node. In this scheme, the BS is the root node of the tree, and then, sequentially, the cell head with the minimum energy consumption is elected to join the tree, until all the cell heads are added to the tree. The advantage of GB-PEDAP is that the use of grid-based tree data aggregation can reduce the energy consumption of nodes, and the energy consumption of GB-PEDAP is more uniform than that of PEDAP. In GB-PEDAP, the depth of the tree structure may be long, and the number of child nodes of the sensor node may be large. The longer the depth of the tree structure, the higher the energy consumption of the farther sensor nodes. In addition, the more child nodes of a sensor node, the higher the energy consumption of its parent node for data transmission. Therefore, GB-PEDAP may cause uneven energy consumption of the sensor nodes.

The schemes mentioned above are mainly to reduce the energy consumption of the sensor nodes or to uniformize the energy consumption of the sensor nodes, so as to prolong the network lifetime. The proposed LB-TBDAS attempts to transmit data from the point of view of load balancing of node energy consumption. This scheme constructs an energy consumption load balancing tree for data aggregation to further improve the lifetime of the WSN.

## 3. The Proposed Scheme

In a WSN, since a very large number of sensors are deployed in the sensing area, the amount of data sensed by the sensor nodes will be very large. If the sensed data are directly transmitted to the BS, the energy of the sensor itself will be quickly exhausted, so that the survival time of the WSN cannot be prolonged. In order to solve these problems of poor network performance, a well-designed data transfer protocol needs to be applied.

We propose a load balancing tree-based data aggregation scheme (LB-TBDAS) in a grid-based WSN. In this scheme, sensors are uniformly deployed in the sensing area at fixed positions, and a grid structure is established in the network area evenly divided into many cells. For transmission, each cell selects the cell head with the highest residual energy. When building a tree structure, the BS is the root node of the tree and LB-TBDAS builds a tree-like path of cell heads according to the constraints of tree construction. In this scheme, each node aggregates data and transmits data to the BS through the tree-like path. LB-TBDAS has three stages: grid construction, tree structure construction, and data aggregation.

### 3.1. Grid Construction

**Figure 1:**
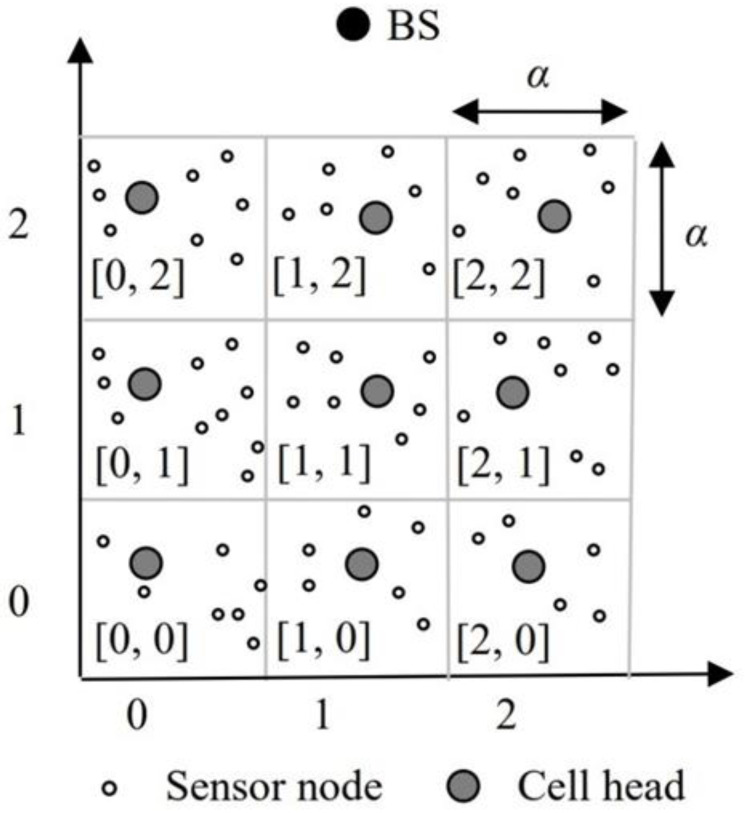
Grid structure.

This paper firstly establishes a grid infrastructure that divides the specific network area into *M* × *N* cells of a grid. Suppose the cell size is α, that is, the cell’s area is α × α. The coordinates of each cell are represented by [*CX*, *CY*]. As shown in Figure 1, we give an example where the network area is partitioned into 3 × 3 cells. The coordinates of cells, from left to right, on the first row are [0, 0], [1, 0], and [2, 0], respectively. The coordinates of cells on the second row are [0, 1], [1, 1], and [2, 1]. The coordinates of cells on the third row are [0, 2], [1, 2], and [2, 2]. The geographic location of each sensor node in the grid is represented by (*x*, *y*). Each node is equipped with a GPS device [18,19] to receive its location information. When the network lifetime begins, each sensor node calculates the coordinates of the cell that it belongs to. Next, the sensor node with the highest residual energy is elected to be the cell head in each cell. When executing each round, each cell reselects the cell head to achieve the purpose of uniform energy consumption.

### 3.2. Tree Structure Construction

**Figure 2:**
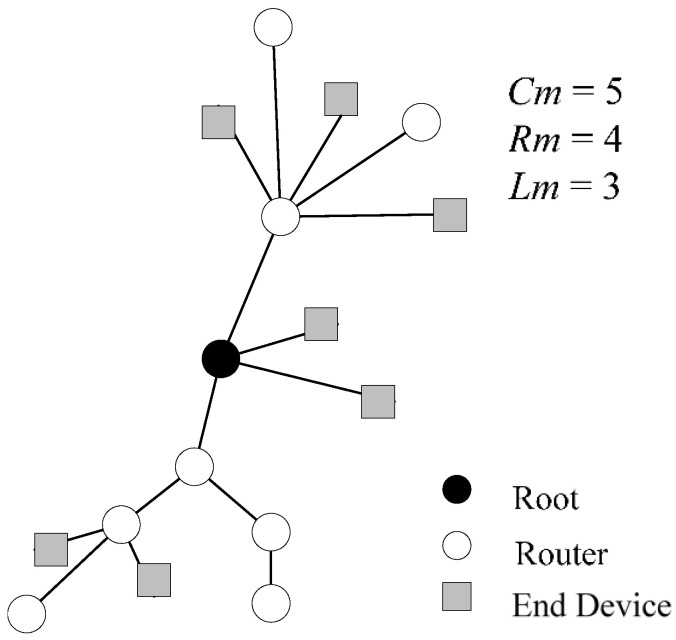
Network architecture of ZigBee.

In the ZigBee network layer protocol [20], a distributed network address allocation algorithm is formulated to allocate network addresses to sensor nodes in WSNs. The network architecture of ZigBee is shown in Figure 2. In the network formation, the ZigBee coordinator defines the maximum number of child nodes of the router *Cm*, the maximum number of child routers of the router *Rm*, and the network depth *Lm*. The child nodes of the router include the other routers and the end devices, so *Cm ≥ Rm*. The address of each device is calculated through *Cm*, *Rm*, and *Lm* to calculate the relevant address parameters, thus the network addresses of the routers and the end devices can be determined [21,22].

PEDAP [16] is a typical tree-based data aggregation scheme based on the minimum energy consumption spanning tree. The scheme calculates the cost of energy consumption for each node by using Equations (1) and (2) [16]. *E_elec_* represents the power consumption for the transmitter circuit or receiver circuit, and *E_amp_* represents the power consumption of the amplifier for data packet transmission. *d_ij_* represents the distance between node *i* and node *j*, and *d_iB_* represents the distance between node *i* and the BS. *Cost_ij_*(*k*) represents the cost for transmitting a packet *k* from node *i* to node *j*, and *Cost_iB_*(*k*) represents the cost for transmitting a packet *k* from node *i* to the BS.

*Cost_ij_*(*k*) = 2 × *E_elec_* × *k* + *E_amp_* × *k* × *d_ij_*^2^ (1)

*Cost_iB_*(*k*) = *E_elec_* × *k* + *E_amp_* × *k* × *d_iB_*^2^ (2)

In the tree establishment of PEDAP, the BS is responsible for serving as the root node of the tree, then the node with the minimum energy consumption is elected to join into the tree, then the process is repeated until all sensor nodes are added to the tree. In each execution round, the leaf nodes of the tree transmit the data to the upper layer of the tree according to the tree-like path for data aggregation, then the process is repeated until the data are transmitted to the BS. PEDAP can reduce the cost required for data transmission and achieve the purpose of reducing the energy consumption of sensor nodes.

This study proposes the LB-TBDAS, which is based on GB-PEDAP [17] by adding constraints on the tree structure to achieve the purpose of load balancing data transmission. In LB-TBDAS, the sensor node with the highest residual energy is elected to be the cell head in each cell. In the tree establishment of LB-TBDAS, the BS is responsible for serving as the root node of the tree, then the node (cell head) which meets the three restrictions, stated later, is elected to join the tree. Then, the nodes (cell heads) are added in sequence in the same way until all the nodes (cell heads) are added to the tree. The proposed LB-TBDAS can avoid the problems of a too long tree depth and too many child nodes (cell heads). Therefore, it can even the energy consumption of nodes to extend the network lifetime.

The node (cell head) *H_i_* is joined into the tree, and the node (cell head) must meet the following conditions:

(a) The node (cell head) does not exist in the tree and has the minimum energy consumption.
(b) The current number of child nodes (cell heads) connected to the node (cell head) *i* is *Cm_i_*, where *Cm_i_* ≤ *Cm*.
(c) The current depth of the node (cell head) *i* is *Lm_i_*, where *Lm_i_* ≤ *Lm*.

In general, the location of the BS will affect the topology of the tree structure, which will also affect the network depth *Lm*. The network depth *Lm* can be determined using Equation (3).

(3) $$Lm=\frac{M+N}{4}$$

**Figure 3:**
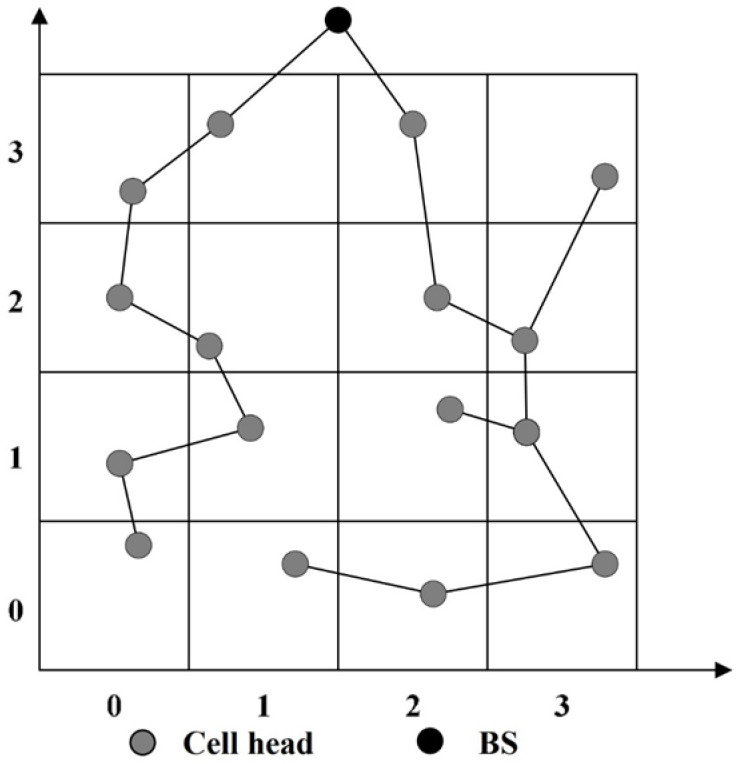
Tree-like path establishment of GB-PEDAP.

**Figure 4:**
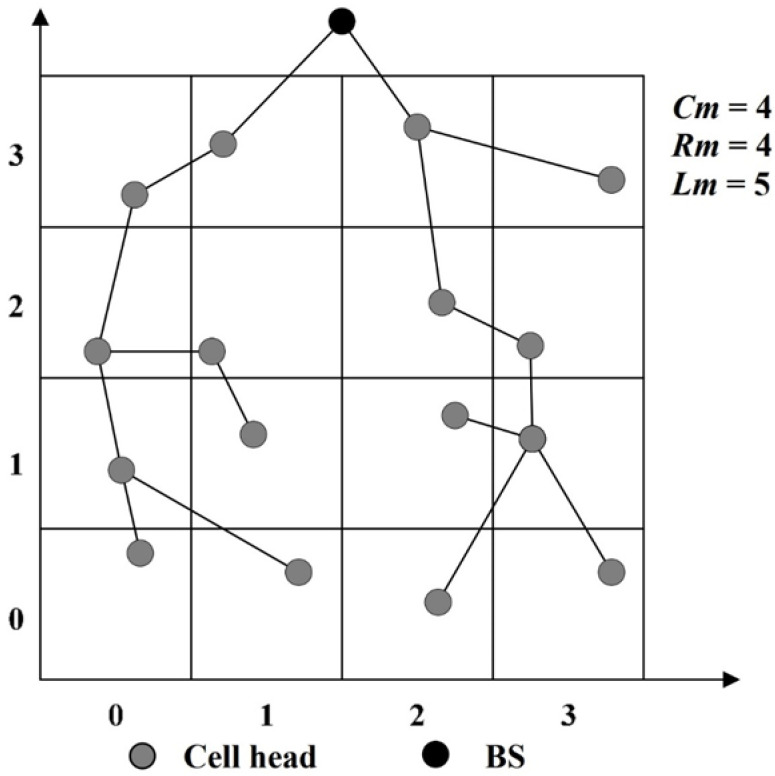
Tree-like path establishment of TBDAS.

In the following, we give an example to discuss the differences between the tree structure of GB-PEDAP and that of the proposed LB-TBDAS. We assume that the BS is located above the sensing area. The tree-like path establishment of GB-PEDAP and LB-TBDAS is shown in Figure 3 and Figure 4, respectively. In Figure 3, the depths of the leaf nodes of the GB-PEDAP tree structure are 7, 7, 5, and 4, respectively, and *Lm* = max (7, 7, 5, 4) = 7. In Figure 4, the depths of the leaf nodes of the LB-TBDAS tree structure are 5, 5, 5, 5, 5, 5, and 2, respectively, and *Lm* = max (5, 5, 5, 5, 5, 5, 2) = 5. Compared with the GB-PEDAP tree structure, the depth of the LB-TBDAS tree structure is more average. Since LB-TBDAS limits the network depth of the tree structure, the number of hops for data transmission is reduced, thus reducing the energy consumption of sensor nodes. In addition, we use the constraint of *Cm*, so that the number of child nodes connected by a node will not be too many, thereby avoiding the problem of hotspots. The tree-like path establishment algorithm of LB-TBDAS is shown in Algorithm 1.
**Algorithm 1:** The tree-like path establishment algorithm of LB-TBDAS.**Step 1:** System initialization(1) Sensor nodes are randomly deployed in the specific network area.(2) The network area is partitioned into *M* × *N* cells of a grid.(3) The sensor node with the highest residual energy is elected to be the cell head in each cell.**Step 2:** Tree initialization(1) The BS is responsible for serving as the root node.(2) The network depth is *Lm* and the maximum number of child nodes (cell heads) is *Cm*.**Step 3:** Tree construction

### 3.3. Data Transmission

When the tree structure is established, the sensing data of sensor nodes are collected and transmitted to their cell head in each cell, and then the sensing data are transmitted to the BS through the tree-like path. For the next execution round, the cell head is re-selected and the tree is re-established; it is processed in the same way. The data aggregation scheme can evenly consume energy, thereby prolonging the network lifetime.

## 4. Simulation Results

In this study, we developed a simulator with MATLAB software. In the simulations, the energy consumption of the sensor nodes in the sensing network adopts the First Order Radio Model [23,24]. When a sensor node does not have enough residual energy for data transmission, the sensor node will be marked as a dead node and the node will no longer transmit data. The network size is 100 m × 100 m and the location of BS is (50, 150). The number of cells is assumed to be 10 × 10 and the initial energy is assumed to be 0.25 J/node. The number of nodes is from 100 to 400 and the packet size is 512 bits. The network depth (*Lm*) ranges from 5 to 10 and the maximum number of child nodes (*Cm*) can be 4 and 7, respectively. *E_elec_* is 50 nJ/bit and *E_amp_* is 100 pJ/bit/m^2^. The simulation parameters of this study are shown in Table 1.

### 4.1. Number of Rounds Versus Node Death Percentages

**Figure 5:**
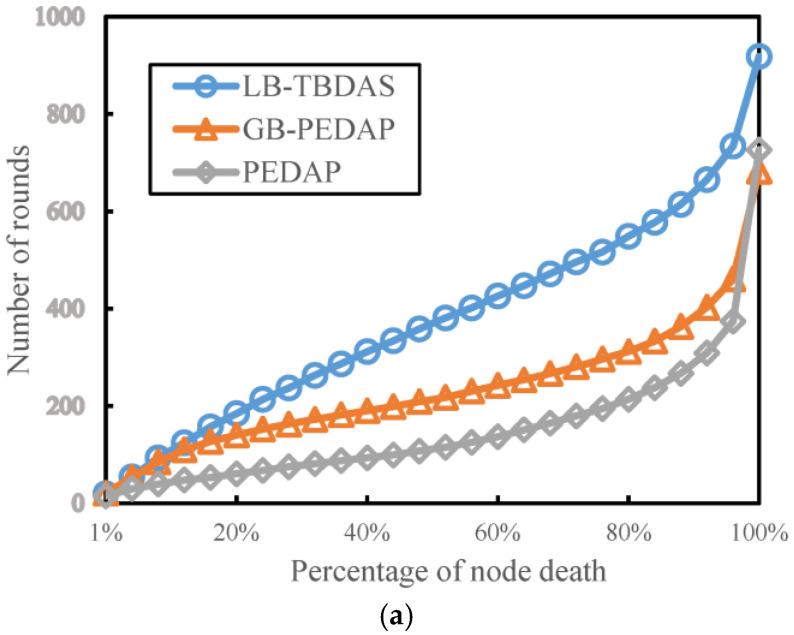

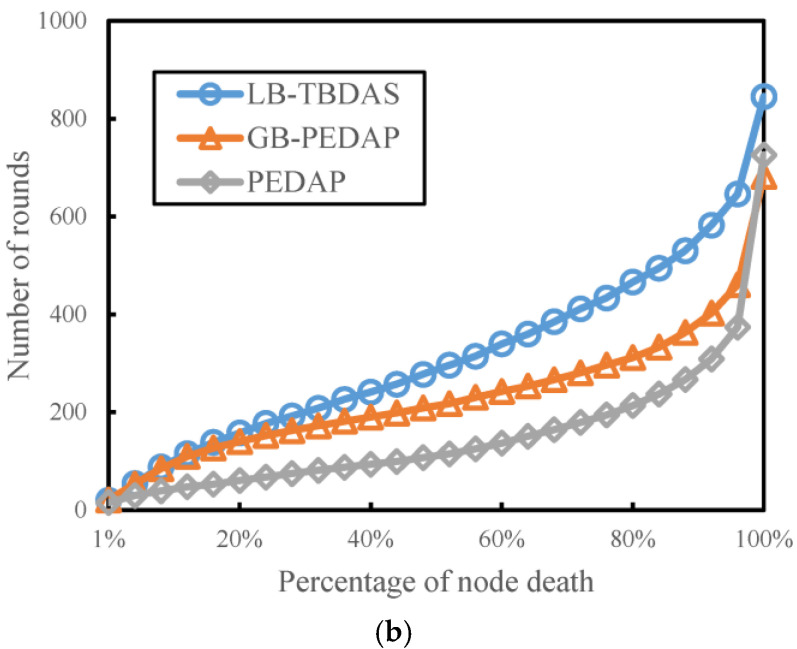
Number of rounds versus node death percentages: (**a**) *Lm* = 5; (**b**) *Lm* = 10.

We study the number of execution rounds of the three schemes at different node death percentages and explore the effect of the LB-TBDAS network depth *Lm*. The number of cells is assumed to be 10 × 10 and the number of nodes is 300. The network depths (*Lm*) are 5 and 10, respectively. The maximum number of child nodes (*Cm*) is 4. We simulated PEDAP, GB-PEDAP, and LB-TBDAS to observe the execution rounds of different node death percentages. In Figure 5a,b, when the node death percentage increases, the number of execution rounds of various schemes also increases. In addition, the LB-TBDAS network depth *Lm* is smaller, and there will be more execution rounds. Overall, the number of execution rounds of LB-TBDAS is better than GB-PEDAP and PEDAP. This is because LB-TBDAS limits the width and depth of the tree-like path structure, which can uniformize the energy consumption of nodes.

### 4.2. Number of Rounds when 50% of Nodes Die versus Number of Nodes

**Figure 6:**
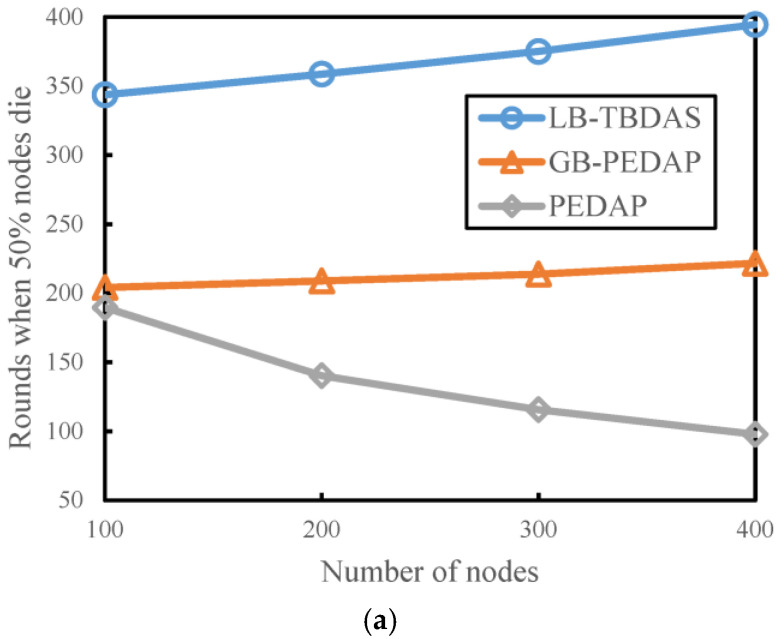

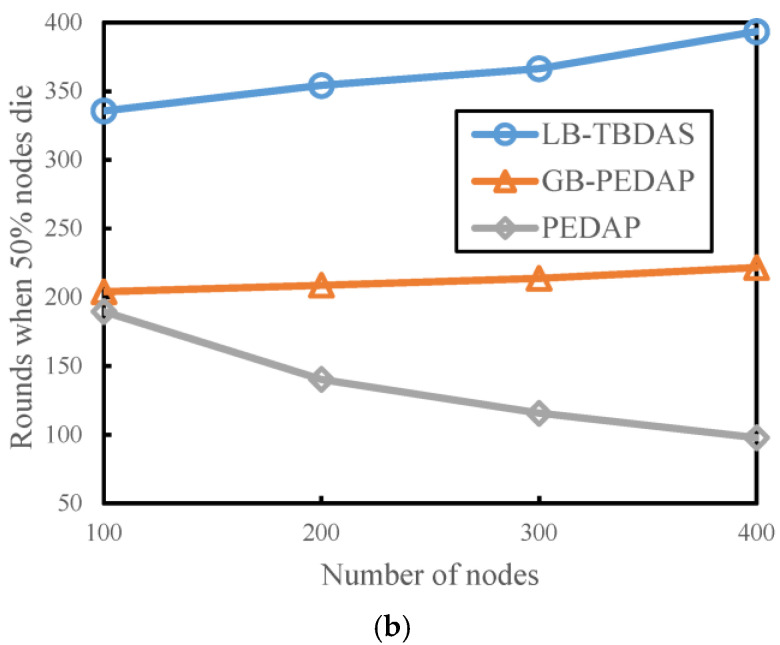
Number of rounds when 50% of nodes die versus number of nodes: (**a**) *Cm* = 4; (**b**) *Cm* = 7.

We observe the number of execution rounds of each scheme when 50% of the nodes die with different numbers of nodes and explore the impact of different values of the maximum number of child nodes (*Cm*) for LB-TBDAS. The number of cells is assumed to be 10 × 10 and the number of nodes is assumed to be 300. The network depth (*Lm*) is 5. The maximum number of child nodes (*Cm*) can be 4 and 7, respectively. In Figure 6a,b, when the number of nodes gradually increases, the execution rounds of LB-TBDAS and GB-PEDAP also increase when 50% of the nodes die, but the execution rounds of PEDAP decrease when 50% of the nodes die. With the same number of nodes, LB-TBDAS executes more rounds than GB-PEDAP and PEDAP when 50% of the nodes die. In LB-TBDAS, as the maximum number of child nodes *Cm* increases, the number of execution rounds increases slightly. When the maximum number of child nodes is larger, the number of hops for data transmission decreases, resulting in a more even load of node energy consumption.

### 4.3. Number of Rounds Versus Depth of Network

**Figure 7:**
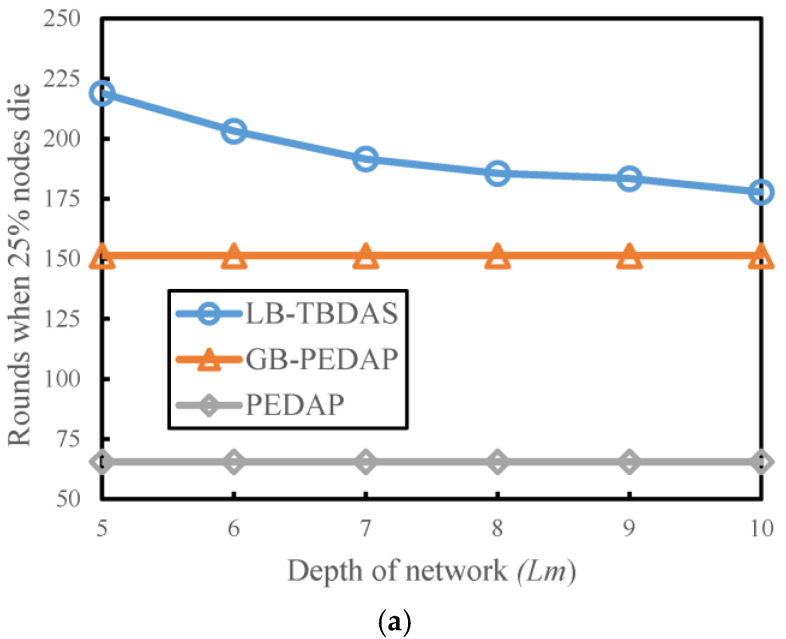

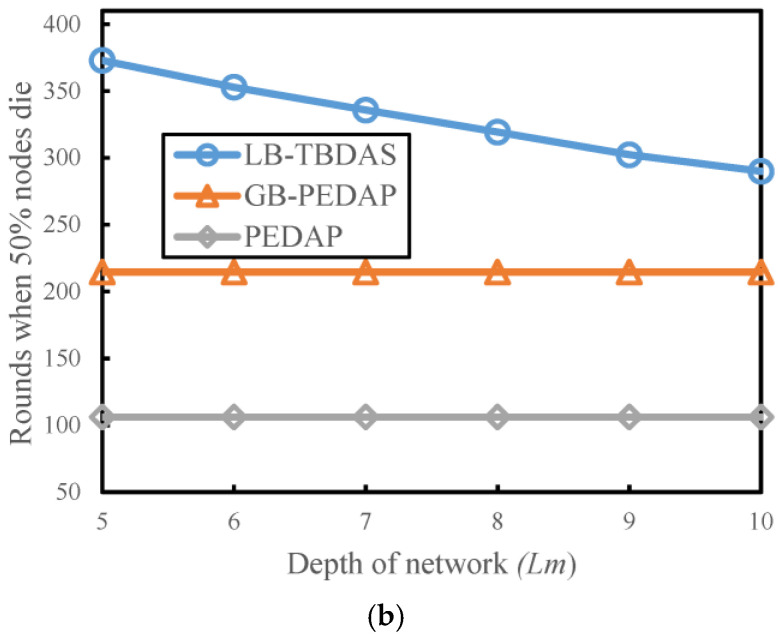
Number of rounds versus depth of network: (**a**) 25% of nodes die; (**b**) 50% of nodes die.

We explore the number of execution rounds of each scheme when 25% and 50% of nodes die at different network depths *Lm*. The number of cells is assumed to be 10 × 10 and the number of nodes is assumed to be 300. The network depth (*Lm*) is from 5 to 10. The maximum number of child nodes (*Cm*) is 4. In Figure 7a,b, when the network depth *Lm* increases, the number of execution rounds of LB-TBDAS at the 25% and 50% node death percentages also increases. As the network depth of LB-TBDAS increases, the number of execution rounds decreases. When the network depth is deeper, the number of hops for data transmission increases, resulting in a more uneven load of node energy consumption.

### 4.4. Total Consumed Energy versus Number of Rounds

**Figure 8:**
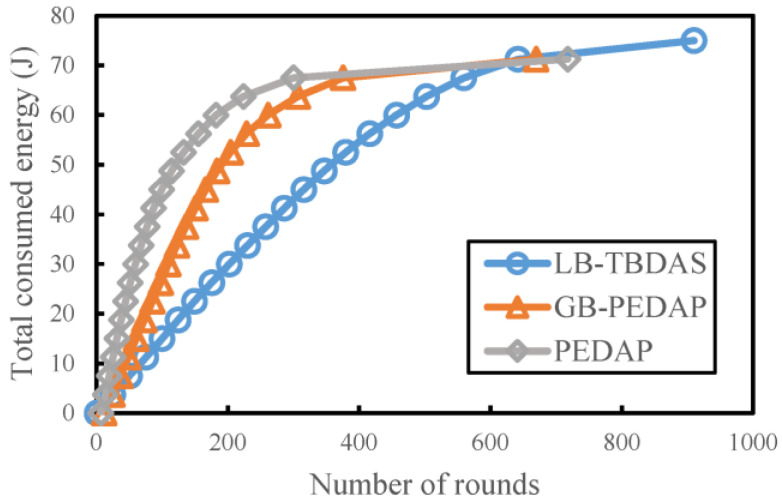
Total consumed energy versus number of rounds.

We study the total energy consumption for sensor nodes in the WSN. The total energy consumption is mainly to observe the energy consumption generated when each node transmits and receives data in each round. The number of cells is assumed to be 10 × 10, the number of nodes is assumed to be 300, and the initial energy is assumed to be 0.25 J. As shown in Figure 8, when the number of execution rounds is gradually increased, the total energy consumption will also increase. The total energy consumption of LB-TBDAS was significantly less than that of GB-PEDAP and PEDAP. This is because a factor considering energy consumption is added to the tree-like path structure of LB-TBDAS, which can reduce the energy consumption of nodes.

### 4.5. Energy Distribution for Sensor Nodes

**Figure 9:**
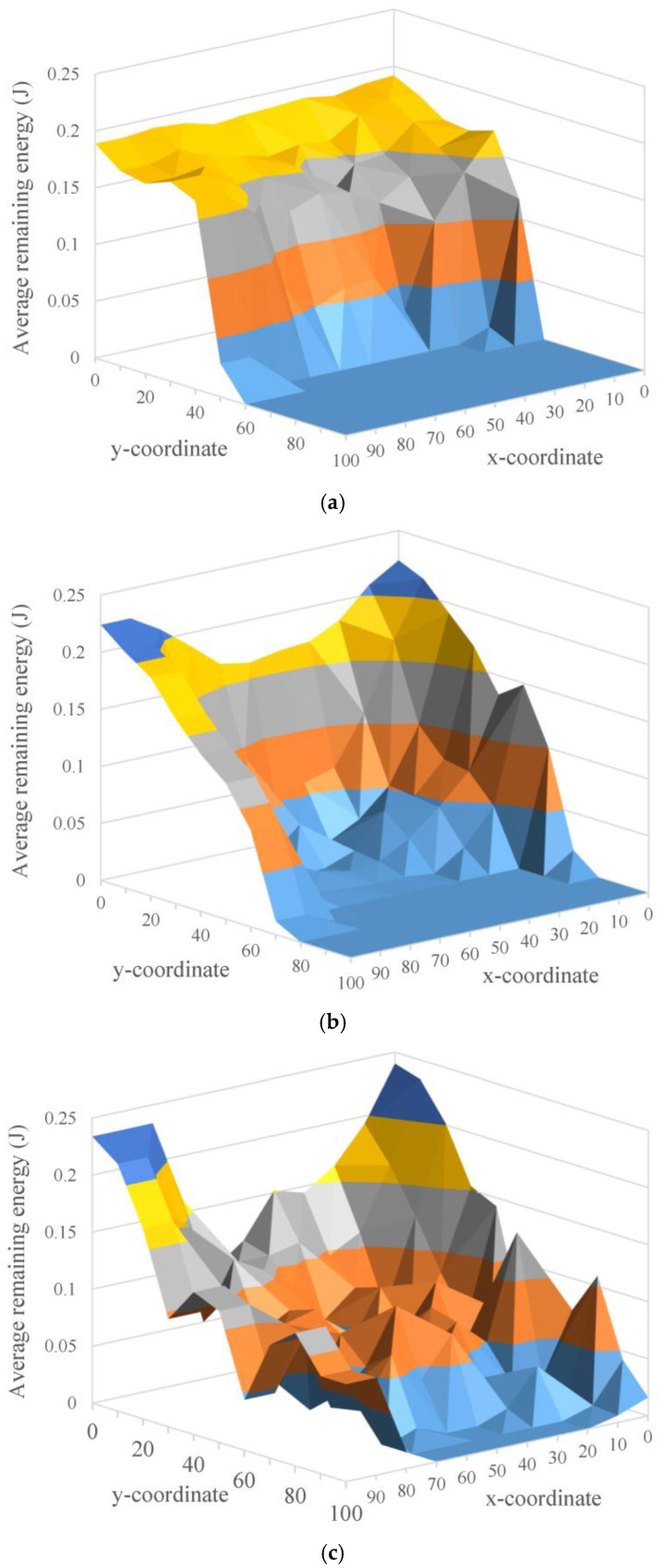
Energy distribution for sensing nodes: (**a**) LB-TBDAS; (**b**) GB-PEDAP; (**c**) PEDAP.

We discuss the energy distribution for sensor nodes in the WSN. We simulate the residual energy distribution of nodes in the sensing area when 50% of the sensor nodes die. In Figure 9a–c, when half of the nodes die, the energy distribution of LB-TBDAS nodes is relatively uniform, and the remaining energy is relatively large; the energy of nodes in the middle area of GB-PEDAP is particularly low and relatively uneven; and PEDAP is completely unevenly distributed in the entire sensing area. This is because LB-TBDAS has the characteristic of load balancing, which can make the energy distribution of nodes more even.

The comparisons of LB-TBDAS, GB-PEDAP, and PEDAP are shown in Table 2. The hierarchical architecture of LB-TBDAS and GB-PEDAP includes two layers and that of PEDAP is a single layer. The data transmission of LB-TBDAS and GB-PEDAP includes direct transmission and tree-path transmission, while the data transmission of PEDAP is tree-path transmission. The energy consumption types of LB-TBDAS, GB-PEDAP, and PEDAP are load balancing, uniform, and general, respectively. The energy efficiency of LB-TBDAS is the best.

In PEDAP, the tree construction does not consider the residual energy of the current sensor nodes, which makes the residual energy distribution very uneven. The GB-PEDAP is a two-layer architecture which builds a grid structure in the sensing area, and then uses Prim’s algorithm to construct an energy consumption uniform tree for data aggregation. The residual energy distribution of GB-PEDAP is more uniform than that of PEDAP. The proposed LB-TBDAS is also a two-layer architecture with a grid which constructs an energy consumption load balancing tree for data aggregation. The residual energy distribution of LB-TBDAS is more even than that of GB-PEDAP and PEDAP.

## 5. Conclusions

In this study, we propose an energy-efficient load balancing tree-based data aggregation scheme (LB-TBDAS) with a grid-based WSN. This scheme uses the minimum spanning tree algorithm to build the tree structure in the grid-based WSN. The proposed LB-TBDAS uses three constraints to construct a tree-like data transmission path with load balancing, and the energy load can be evenly dispersed. Simulation results show that the average remaining energy of LB-TBDAS is significantly better than that of GB-PEDAP and PEDAP. The proposed LB-TBDAS extends over 100% of the network lifetime compared to GB-PEDAP and PEDAP. The proposed LB-TBDAS can effectively reduce the energy consumption of sensor nodes, thereby prolonging the network lifetime.

## Figures and Tables

**Table 1:** Simulation parameters.

Parameters	Values
Network area	100 m × 100 m
Location of BS	(50, 150)
Initial energy	0.25 J/node
Number of cells	10 × 10
Number of sensor nodes	100–400
Packet size	512 bits
Network depth (*Lm*)	5–10
Maximum number of child nodes of the node (*Cm*)	4, 7

**Table 2:** The comparisons of LB-TBDAS, GB-PEDAP, and PEDAP.

Protocol	LB-TBDAS	GB-PEDAP	PEDAP
Hierarchical architecture	two layers	two layers	single layer
Data transmission structure	direct and tree	direct and tree	tree
Type of energy consumption	load balancing	uniform	general
Energy efficient	very high	high	low

## Data Availability

Not applicable.
